# Aluminum–Silica Core–Shell Nanoparticles via Nonthermal Plasma Synthesis

**DOI:** 10.3390/nano15030237

**Published:** 2025-02-04

**Authors:** Thomas Cameron, Bailey Klause, Kristine Q. Loh, Uwe R. Kortshagen

**Affiliations:** 1 Department of Mechanical Engineering, University of Minnesota, Minneapolis, MN 55455, USA; camer299@umn.edu; 2 Department of Chemical Engineering and Materials Science, University of Minnesota, Minneapolis, MN 55455, USA; klaus123@umn.edu (B.K.); loh00014@umn.edu (K.Q.L.)

**Keywords:** nonthermal plasma, dusty plasma, metal nanoparticles, aluminum nanoparticles, core–shell, in-flight synthesis

## Abstract

Aluminum nanoparticles (Al NPs) are interesting for energetic and plasmonic applications due to their enhanced size-dependent properties. Passivating the surface of these particles is necessary to avoid forming a native oxide layer, which can degrade energetic and optical characteristics. This work utilized a radiofrequency (RF)-driven capacitively coupled argon/hydrogen plasma to form surface-modified Al NPs from aluminum trichloride (AlCl_3_) vapor and 5% silane in argon (dilute SiH_4_). Varying the power and dilute SiH_4_ flow rate in the afterglow of the plasma led to the formation of varying nanoparticle morphologies: Al–SiO_2_ core–shell, Si–Al_2_O_3_ core–shell, and Al–Si Janus particles. Scanning transmission electron microscopy with a high-angle annular dark-field detector (STEM-HAADF) and energy-dispersive X-ray spectroscopy (EDS) were employed for characterization. The surfaces of the nanoparticles and sample composition were characterized and found to be sensitive to changes in RF power input and dilute SiH_4_ flow rate. This work demonstrates a tunable range of Al–SiO_2_ core–shell nanoparticles where the Al-to-Si ratio could be varied by changing the plasma parameters. Thermal analysis measurements performed on plasma-synthesized Al, crystalline Si, and Al–SiO_2_ samples are compared to those from a commercially available 80 nm Al nanopowder. Core–shell particles exhibit an increase in oxidation temperature from 535 °C for Al to 585 °C for Al–SiO_2_. This all-gas-phase synthesis approach offers a simple preparation method to produce high-purity heterostructured Al NPs.

## 1. Introduction

Aluminum is an earth-abundant, nontoxic metal fuel that can be readily processed into nanoparticles using ball milling, exploding wire, vapor condensation, and wet chemical methods [1,2,3,4,5]. Al NPs are extremely reactive and energy dense [6]. They are applied in many capacities with notable examples as a plasmonic material owing to their strong and size-tunable resonant behavior [7,8,9,10] and in energetic compounds, including thermites and propellants [11,12,13,14,15]. Recent research has focused on improving the performance of Al-containing nano-thermite mixtures, where the fuel particle diameter is <100 nm, to take advantage of size-dependent energetic characteristics [16,17,18,19]. Melting, ignition temperature, and reaction rate are improved for Al NPs compared to their conventional micron-sized counterparts [20,21,22]. Nanoscale mixtures can also maximize reaction kinetics by placing the fuel and oxidizer in nanoscale proximity with structures like core–shell NPs. This reduces the diffusion distance to speed up the reaction.

A drawback of many Al NP synthesis techniques is their inability to mitigate the formation of a native oxide layer (Al_2_O_3_) on the surface. The surface characteristics of Al NPs directly impact their behavior [23]. Typically, forming a 2–6 nm thick native oxide layer (Al_2_O_3_) results in a deteriorated performance of Al NPs since the native oxide layer is not reactive and does not contribute to the heat released to the reaction, thus limiting it. This limits the Al NPs’ efficacy when applied to thermite mixtures [24]. Surface modification to reduce or replace the native oxide layer can mitigate some of the downsides of Al NPs by preserving more active Al fuel by volume [25,26,27]. One example is the exploding wire preparation of Al–silica (SiO_2_) core–shell particles, which were experimentally reported to have favorable energetic properties but a wide size distribution [2]. SiO_2_ as a shell material also improves the stability of Al–SiO_2_ core–shell particles, as shown by Renard et al. [5].

A fundamental understanding of the formation of core–shell particles has been established through recent mesoscopic scale density functional theory (DFT) modeling efforts [28]. In the case of InAlN nanorods, DFT and phase-field modeling (PFM) were used to predict the parametric dependence of the system on the phase separation, core–shell interface, morphology, and composition of the resulting nanomaterials. DFT PFM generally indicates that lower interfacial energy values are obtained for interphase interfaces [28]. Therefore, the spontaneous formation of core–shell structures can be thermodynamically favorable. The formation of Janus and alloyed nanoparticles can similarly be explained through molecular dynamics (MD) simulations [29]. MD modeling has shown that structural evolution is strongly dependent on the temperature at which the particles form [30]. Modeling particle formation in plasmas becomes much more difficult due to transient excited and ionized species in a highly reactive environment driven by high-energy electron impacts. Recent work in this field has focused on modeling to understand the nucleation and growth of semiconductor materials, such as Si in plasma [31,32,33,34]. Although out of scope for this work, future advancement in modeling these complex systems is promising to contextualize the experimental results, inform future experimentation, and enable more precise control over engineered nanomaterials from nonthermal plasma synthesis.

Experimentally, nonthermal plasma synthesis of Al NPs and other energetic materials has been shown to produce narrow size distributions with a small average particle size that exhibit desirable size-dependent energetic behavior [16,17,35,36,37,38]. This synthesis technique allows for the in-flight formation and coating of nanoparticles in a single step. Low-pressure processes such as this are particularly desirable for the formation of non-oxide passivated core–shell NPs because the particles can be coated before they are exposed to the atmosphere. Preventing surface oxidation via SiO_2_ shell formation may benefit the optical properties of Al nanoparticles. Colloidally formed Al–SiO_2_ core–shell particles have been shown to have favorable UV plasmonic resonance and broadband absorption [5]. A computational study by Yun-Fei et al. concluded that Al–SiO_2_ core–shell particles cause a redshift of the localized surface resonance peak. Their simulations showed that Al–SiO_2_ core–shell structures would improve the efficacy of organic solar cells [39]. Another computational study demonstrated the enhancement of the optoelectronic behavior of thin-film solar cells when Al NPs coated with a thin layer of SiO_2_ were added to the system [10].

Recently, magnesium–Si-SiO_2_ core–shell NPs were synthesized using nonthermal plasma and silane gas (SiH_4_) [36]. This heterostructure enhanced the energetic characteristics of the material by reducing the ignition temperature by 29%, from ~740 °C to ~520 °C. The enhancement was due to the reduction in the native magnesium oxide layer and the beneficial exothermic alloying reactions between magnesium and silicon that occur around 400 °C. Motivated by the promise of dusty plasma synthesis and the success of prior studies [40], we demonstrate here the gas-phase synthesis of Al–Si NP heterostructures. We identify how changes to the experimental plasma parameters affect the composition and morphology of the resulting nanomaterial.

We focus on Al NPs modified with SiO_2_. In the case of Al–SiO_2_ core–shell NPs, the amorphous Si (a-Si) surface oxidizes when exposed to the atmosphere to form SiO_2_. Importantly for energetic applications, this shell material will react with Al at approximately 1200 °C in an exothermic reaction, as shown in Equation (1) [41,42]. By passivating the Al nanoparticle surface with SiO_2_, Al can directly react with oxygen after it melts and diffuses through the shell at lower temperatures [23]. Al oxidizes exothermically, as shown in Equation (2).

(1) $$4Al+3SiO_{2}\to 2Al_{2}O_{3}+3Si\;\;\;\;\Delta H॰=-2.15\frac{kJ}{g}$$

(2) $$4Al+3O_{2}\to 2Al_{2}O_{3}\;\;\;\;\Delta H॰=-31\frac{kJ}{g}$$

One limitation of conventional thermite mixtures is the homogeneity and material proximity of the fuel and oxidizer mixture [11,43]. Plasma synthesis can overcome this by forming NPs with a precisely tuned fuel-to-oxidizer ratio within a favorable nanoscale structure. The formation of complexly structured particles, such as core–shells or Janus NPs, in a plasma with purely gas-phase precursors occurs rapidly in the order of the residence time of the particles within the plasma (ms). Core–shell particles with a nonporous passivating layer are the most desirable structure to target for synthesis because they put materials in nanoscale proximity and have a conformal heterogeneous layer to limit oxygen diffusion to the core. This avoids a known drawback of particle mixtures containing nano-Si for energetics, which have the potential to undergo the process of small particle sintering during combustion, which is parasitic to the energetic reactions [44]. Therefore, phase-separated mixtures simply containing Si NPs and Al NPs were considered undesirable.

Understanding how to experimentally control the synthesis process will provide a unique opportunity to apply a continuous nonthermal plasma-based process to produce surface-modified Al NPs. This work demonstrates that this synthesis technique has the promise to form morphology tunable, passivated Al NPs in an in-flight process.

## 2. Materials and Methods

### 2.1. Experimental Setup

**Figure 1:**
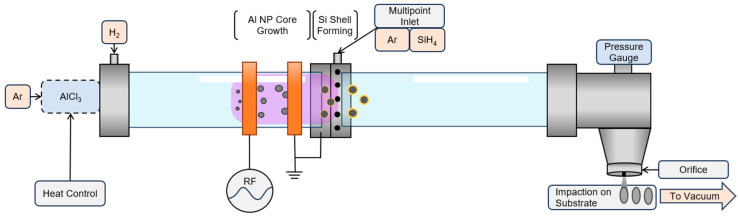
Reactor schematic of the CCP reactor showing the relative positions of the copper ring electrodes in orange, the plasma region in purple, and the gas inlets for Ar/AlCl_3_, H_2_, and dilute SiH_4_ in light orange.

The experimental setup presented in this work was adapted from that presented in previous publications [37,38]. A schematic of this setup is presented in Figure 1. Aluminum trichloride (AlCl_3_, anhydrous powder, 99.999% trace metals basis, Sigma-Aldrich, St. Louis, MO, USA) was used as the precursor to form Al NPs. AlCl_3_ was loaded into a heated stainless-steel vessel to sublimate at 130 °C. The AlCl_3_ vapor was then mixed with the carrier gas, argon (Ar) (Airgas, AR UPC300, 99.9993%), and flowed into the plasma region of the reactor. Hydrogen (H_2_) was introduced into the reactor to serve as an AlCl_3_ reductant and chlorine scavenger, aiding in the crystallization of the Al NPs [38]. The H_2_ gas flow rate was constant at 100 standard cubic centimeters per minute (sccm). The Ar carrier gas flow rate for the AlCl_3_ precursor was constant at 75 sccm.

After the Al core forming region, downstream of the plasma, 5% SiH_4_ diluted in Ar was introduced into the reactor using a radially symmetric 16-point inlet near the plasma afterglow. The reactor had two 25 cm long quartz tubes joined by Swagelok Ultra-Torr fittings. The inner diameter of the quartz reactor tube was 36 mm. After the Si shell forming region, the particles were accelerated through a 0.5 mm slit orifice and collected via impaction onto substrates for characterization [45].

An RF-driven capacitively coupled plasma (CCP) was ignited with an RF power supply (AG 0313 generator and amplifier, T&C Power Conversion, Inc., Rochester, NY, USA) connected to an in-house fabricated matchbox. The input power varied from 20 to 60 W. Externally placed copper ring electrodes with a 38 mm inner diameter were placed 35 mm apart. The powered electrode was 40 mm from the multipoint SiH_4_ inlet. One important consideration for the success of forming core–shell particles was controlling the electrode distance from the dilute SiH_4_ inlet position since RF power density is known to influence Si particle clustering behavior [46]. For this experimental setup, it was found that a powered electrode distance of 40 mm from the dilute SiH_4_ inlet resulted in reproducible samples.

The combined gas flow rate was 203 sccm with a constant nominal operating pressure of 5.4 Torr. The total gas flow rate was minimized to increase the residence time of the Al NPs in the plasma to about 140 ms, which promotes in-flight surface modification by extending the exposure time NPs have in the plasma coating region. An additional Ar source was mixed with the dilute SiH_4_ to keep the pressure in the reactor constant as the SiH_4_ flow was adjusted. This diluting Ar flow was 20–28 sccm, while the dilute SiH_4_ flow was adjusted between 0 and 8 sccm, keeping the total gas flow and pressure nearly constant in the reactor. The conditions under which both Al NPs can be formed and Si surface treatment occurs were balanced to achieve core–shell heterostructures.

Hydrogen-terminated crystalline Si particles were synthesized to compare their oxidative behavior with Al–SiO_2_ NPs. Si NPs were produced in a separate experimental setup using a similar synthesis methodology to that described in the PhD thesis of Rebecca Anthony [47] (pp. 98–100). In this case, the Ar and SiH_4_ (5% in He) flowed through an RF plasma at 65 W with flow rates of 30 sccm and 14 sccm, respectively. H_2_ was injected in the plasma afterglow at a flow rate of 100 sccm to further terminate the surface with hydrogen. Crystalline Si (c-Si) particles were formed and grown in the plasma region and were then collected by impaction on a glass substrate (optical microscope slide).

### 2.2. Material Characterization

Transmission electron microscopy (TEM), scanning transmission electron microscopy (STEM), and energy-dispersive X-ray spectroscopy (EDS) measurements were performed on a Thermo Scientific Talos F200x electron microscope (Thermo Scientific, Waltham, MA, USA). TEM, STEM, and EDS were performed at 200 kV accelerating voltage. For STEM/EDS imaging, a high-angle annular dark-field (HAADF) detector was used alongside a Super-X EDS system (Thermo Scientific, Waltham, MA, USA). Samples were first sonicated in methanol and then drop-cast onto 3 mm diameter lacey carbon-supported copper TEM grids from Pacific Grid-Tech (Pacific Grid-Tech, San Francisco, CA, USA). Particle dimensions were manually measured using the open-domain scientific image analysis software ImageJ (version 1.53k) for sizing. Between 136 and 205 particles were measured for each condition, where the geometric mean diameter is reported with geometric standard deviations.

X-ray diffraction (XRD) measurements were taken with a Bruker D8 Discover diffractometer (Bruker AXS, Madison, WI, USA) equipped with a cobalt (Co) Kα source (wavelength (λ) = 1.79 Å) [38]. Samples were deposited by impaction onto glass slides. The XRD patterns were converted to copper (Cu) Kα wavelengths (λ = 1.54 Å) for data analysis, which was performed using the Material Data Incorporated Jade 8.0 software package.

Fourier-transform infrared spectroscopy (FTIR) was performed with a Bruker Alpha FTIR (Bruker Optics, Billerica, MA, USA) with a diffuse reflectance (DRIFTS) accessory. The FTIR spectrometer was located in a glovebox with a nitrogen atmosphere. Samples were deposited via impaction in a thin layer on gold-coated Si wafer substrates. Spectra collection, background correction, and min–max normalization were performed using OPUS software (version 8.7.10).

X-ray photoelectron spectroscopy (XPS) was performed with a PHI VersaProbe III XPS and UPS system (Physical Electronics Inc., Chanhassen, MN, USA) with a monochromatic Al Kα anode X-ray source (photon energy = 1486.6 eV) and a hemispherical analyzer. Samples were deposited by impaction onto glass slides. XPS survey scans were taken with a pass energy of 280 eV. The binding energy of C 1s at 284.8 eV was used as a reference. XPS high-resolution scans were taken with a pass energy of 55 eV. High-resolution scans were shifted with an internal reference of SiO_2_ at 103.4eV in the 20 W and 40 W high-resolution spectra and 103.2 eV in the 60 W spectrum [48]. Atomic percentages were calculated, and peak fitting was performed using PHI’s Multipak software v9.0.

Thermogravimetric analysis (TGA) and differential scanning calorimetry (DSC) were performed using a TA Instruments SDT-Q600 thermal analyzer (TA Instruments, New Castle, DE, USA) for simultaneous measurements of TGA and DSC. The weight percent presented in this work was calculated based on the initial sample mass measured by the instrument when the heating started. The heating rate was set to a constant 5 °C per minute ramp from 50 to 1100 °C. The purge gas was synthetic air, flowing at 20 mL/min. Higher purge flow rates or heating rates induced sample ignition. A total of 90 μL alumina crucibles from Thermal Support (Thermal Support, Hayesville, NC, USA) were used for the measurements. Since the particles were collected via impaction, the piles were crushed to disperse the NPs and improve the exposed surface area.

All samples were exposed to the air very briefly while being loaded into the XPS and TEM. XRD measurements were performed in ambient air. For TGA/DSC measurements, the samples were exposed to the atmosphere for sample preparation and to avoid autoignition but were otherwise handled air free.

## 3. Results and Discussion

### 3.1. STEM and EDS Analysis

**Figure 2:**
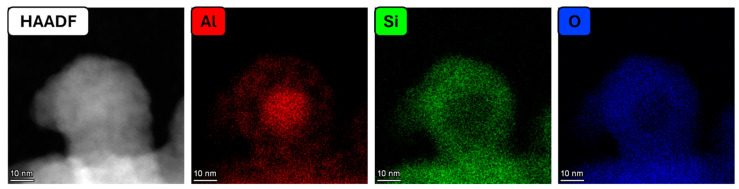
A STEM-HAADF and EDS elemental map for an Al–SiO_2_ core–shell particle formed at 50 W and 4 sccm dilute SiH_4_.

High-resolution STEM HAADF imaging and EDS mapping were performed to understand the structure and composition of the particles. In addition, line profile and area profile EDS analyses were performed to visualize the spatial distribution of elements within representative particles. An elemental map for an Al–SiO_2_ core–shell particle is shown in Figure 2, showing a core–shell structure. Additional elemental maps of Al–SiO_2_ are presented in the supporting information in Figure S1.

The HAADF image and elemental map in Figure 2 show a spherical particle where Al is primarily localized in the core. This was also the case for the other samples depicted in Figure S1. The Si signal correlated closely with that of O, and both were limited to the particles’ periphery.

**Figure 3:**
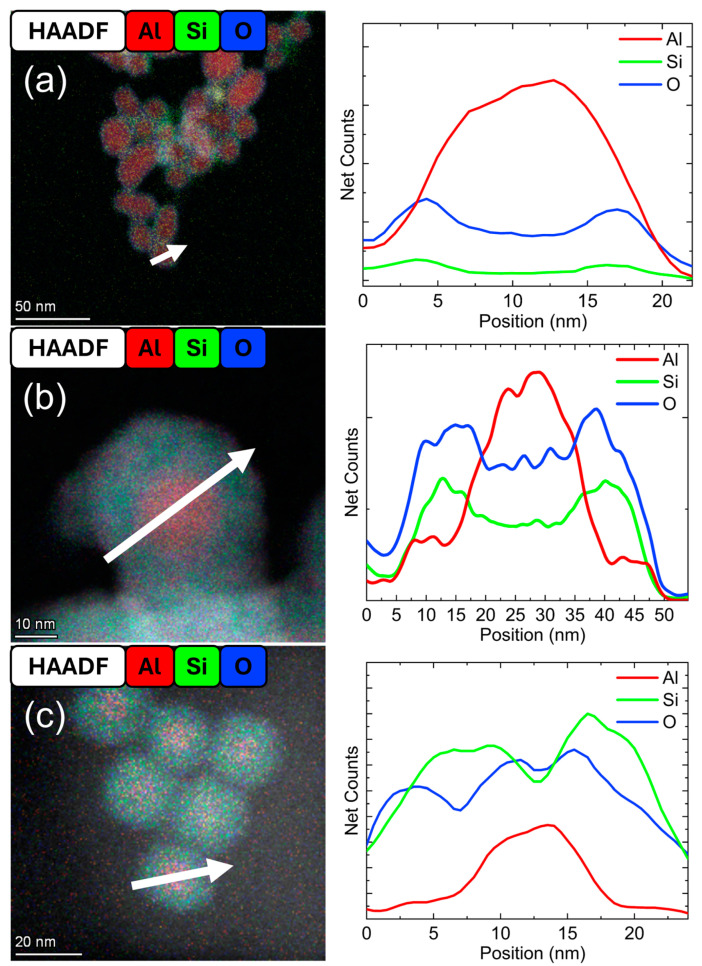
STEM-HAADF and elemental map color mix with EDS line profiles (based on the elemental map with a 4-pixel averaging prefilter) for Al–SiO_2_ core–shell particles formed at (**a**) 20 W and 4 sccm dilute SiH_4_, (**b**) 50 W and 4 sccm dilute SiH_4_, and (**c**) 60 W and 3 sccm dilute SiH_4_. The white arrows indicate the location and direction of the line profiles.

Figure 3 contains composite mapping images formed by overlaying the elemental maps for core–shell particles synthesized for increasing RF powers while keeping the flow rate of dilute SiH_4_ constant at 4 sccm in the 20 W and 50 W samples. The flow rate of dilute SiH_4_ was reduced to 3 sccm in the 60 W sample, which was necessary to obtain a sample of primarily core–shell particles. Line profiles were generated across the diameter of a single particle, indicated by a white arrow, to represent the distribution of Al, Si, and O. In all line profiles, a four-pixel prefilter was applied. The Cl signal was negligibly small in the line profiles in all cases, indicating sufficient AlCl_3_ reduction.

In Figure 3a, for an RF power of 20 W, the Si and O signals were strongly correlated with a low intensity across the line profile. This indicated a thin-shelled Al–SiO_2_ core–shell structure with a shell thickness of ~2 nm. For these conditions, the size distribution had a geometric mean diameter (μ_g_) of 20 nm with a geometric standard deviation (σ_g_) of 1.31 (N = 205). When the RF power was increased to 50 W with a constant flow rate of 4 sccm dilute SiH_4_, thicker-shelled particles were observed, as shown in Figure 3b. The average shell thickness was 6 nm. Here, μ_g_ was 31 nm with σ_g_ of 1.28 (N = 204), indicative of a nearly monodisperse sample. The Al signal was most prominent in the particle’s core, although a relatively low-intensity signal of Al in the shell was also detectable. This may be indicative of Al diffusion through a porous SiO_2_ surface. In Figure 3c, at 60 W with 3 sccm dilute SiH_4_, the Si signal across the line profile was strong, and a thick Al–Si core–shell structure was observed. The shell thickness was approximately equal to the core diameter of 8 nm. The size distribution of this sample showed μ_g_ of 26 nm with σ_g_ at 1.32 (N = 132). The TEM images and corresponding size distribution plots can be found in the supporting information in Figure S2.

As the RF power was increased from 20 to 60 W, the Si-containing shell thickened. However, the effect of the dilute SiH_4_ flow rate must also be considered. As the flow rate of dilute SiH_4_ was increased to 4 sccm and beyond at an input power of 60 W, either preferential nucleation of c-Si NPs or phase separation of NPs was observed in some samples. This resulted in samples that were primarily uncoated Al NPs mixed with c-Si NPs. Therefore, for heterogeneous surface growth to be preferred in this system, the consumption and formation of Si-H_x_ radicals in the plasma must be balanced. This balance can be achieved by influencing the SiH_4_ precursor dissociation rate by controlling both the power and precursor flow rate simultaneously. This aligns with previously reported observations in the nonthermal plasma synthesis of Ge-Si core–shell nanocrystals [40].

**Figure 4:**
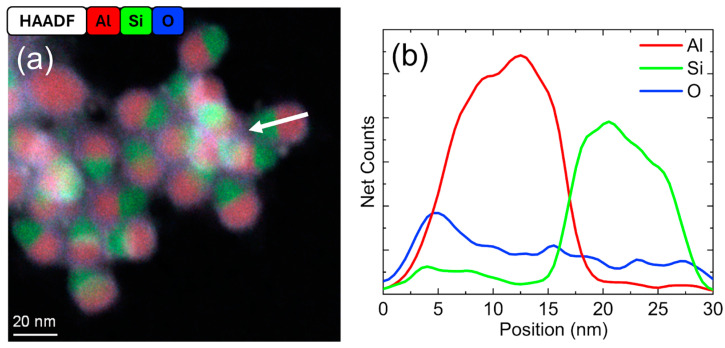
(**a**) Elemental map with (**b**) EDS line profile (based on the elemental map with a 4-pixel averaging prefilter) for Al–Si Janus particles deposited at 20 W with 8 sccm dilute SiH_4_. The white arrow indicates the location of the line profile.

The EDS elemental map in Figure 4a shows particles composed of separate phases of Al and Si that were obtained for significantly larger flow rates of dilute SiH_4_. Such particles are typically called “Janus particles”. As shown in the line profile in Figure 4b, each particle had a thin oxide shell on the surface but not between the two phases. Si and Al existed in individual ellipsoidal particles that were phase separated and oxidized only at the surface. Individual elemental maps showing the HAADF image, Al, Si, and O for this can be found in Figure S3. Selective heating due to energetic surface recombination reactions is known to induce the crystallization of Si nanoparticles in plasma. This system demonstrated similar behavior to other nonthermal plasma studies where RF power caused Si crystallization [35,49].

**Figure 5:**
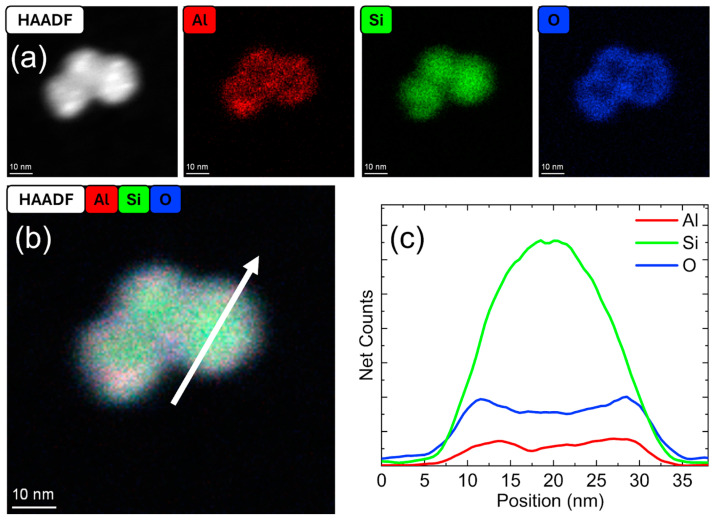
(**a**,**b**) Elemental maps with (**c**) EDS line profile (based on the elemental map with a 4-pixel averaging prefilter) for Si–Al_2_O_3_ particles formed at 40 W and 8 sccm dilute SiH_4_. The white arrow indicates the location and direction of the line profile.

For large flow rates of dilute SiH_4_, Si–Al_2_O_3_ core–shell NPs were observed, as shown in Figure 5. According to the elemental maps in Figure 5a,b and the line profile plot in Figure 5c, the Si signal was localized in the core of the structure. Signals for Al and O overlapped and appeared to form a thin shell. This indicated a Si core surrounded by a thin layer of oxidized Al. The conditions needed to form this structure were 40 W of RF power and a dilute SiH_4_ flow rate of 8 sccm; such structures were also observed at 60 W and 6 sccm dilute SiH_4_. This implied that under the higher power and dilute SiH_4_ flow rate conditions studied, Si NPs are preferentially nucleated, and the Al NPs are either not formed or evaporate within the plasma to coat the Si NPs. The ability to form core–shell particles with Si cores was an unexpected finding in this work that provides a potential future direction for studying a wider range of tunable heterostructures that can be achieved from all-gas-phase nonthermal plasma synthesis. The identification of these structures demonstrates the flexibility and versatility of this plasma-based synthesis technique in the Al–Si system.

One significant outcome of this work was the ability to control the composition of core–shell particles and tune the shell thickness, primarily indicated by the ratio of Al to Si. The composition from EDS is represented in Table 1. Note that carbon is present from the carbon-coated TEM grid.

To summarize, three distinct heterostructures were identified: (1) Al–SiO_2_ core–shell particles, as shown in Figure 2 and Figure 3, (2) Janus Al–Si particles in Figure 4, and (3) Si–Al_2_O_3_ core–shell particles in Figure 5. Overall, the Al:Si ratio and the type of heterostructure were tunable with the RF power and dilute SiH_4_ flow rate. For the conditions described in Figure 3a,c, the Al:Si ratio can be tuned over nearly two orders of magnitude from approximately 0.2–9.8. The Al:Si ratio was maximized in this study at 20 W and 4 sccm dilute SiH_4_. Unmodified Al NPs comprised most of the sample at dilute SiH_4_ flow rates < 2 sccm or RF powers below 20 W; core–shell particles were a minority fraction in those samples. Janus particles and Si–Al_2_O_3_ core–shell particles were observed and reported in Table 1, but further experimentation to tune the composition of these particle structures was beyond the scope of this work. The surface of Al–SiO_2_ NPs appeared to be partially or completely passivated, depending on the shell thickness.

### 3.2. XRD Analysis

**Figure 6:**
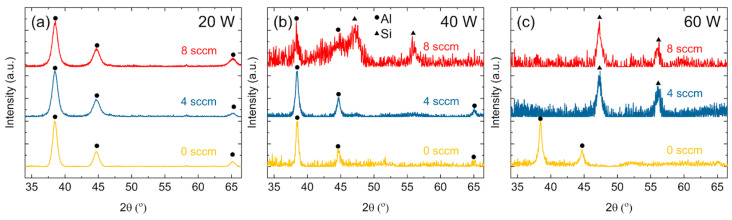
XRD patterns for samples at (**a**) 20 W, (**b**) 40 W, and (**c**) 60 W and 0, 4, and 8 sccm dilute SiH_4_ flow rate.

Whereas STEM-EDS focused on single particles, XRD patterns are presented in Figure 6 to indicate the crystalline phases in the bulk of the sample. In Figure 6a, for NPs synthesized at 20 W, all patterns indicate FCC Al peaks; the peak positions are identified with solid circles at 38.45° (111), 44.63° (200), and 65.07° (220) according to JCPDS No. 04-0787 for FCC Al.

As both the RF power and dilute SiH_4_ flow rate increase, the composition of crystalline phases in the sample changes, and Si peaks become detectable. The peaks for Si are identified with solid triangles at 47.26° (220) and 55.09° (311) according to JCPDS No. 27-1402. In the 40 W case in Figure 6b, c-Si and FCC Al were observed to exist simultaneously at 8 sccm dilute SiH_4_. This indicated phase separation between Si and Al NPs in the bulk of the sample, which correlated with findings from TEM where Si–Al_2_O_3_ core–shell NPs existed in the presence of unmodified Al and Si particles. At 4 sccm, the pattern had only Al peaks. Based on the trends observed from STEM-EDS, the samples deposited at this condition were primarily Al–SiO_2_ core–shell particles. In the 60 W case, preferential c-Si NP formation was observed above 4 sccm dilute SiH_4_. Although some Al NPs were likely present, they were not detectable in the patterns shown in Figure 6c. This indicated that there was competing nucleation between Al and Si NPs. Both material precursors were decomposed in the plasma, but as the RF power or Si concentration was increased, Al NPs did not form and crystallize in detectable quantities. Si–Al_2_O_3_ particles were also observed at 60 W and 4 sccm dilute SiH_4_. However, at 60 W and 8 sccm dilute SiH_4_, the sample was mainly c-Si.

**Figure 7:**
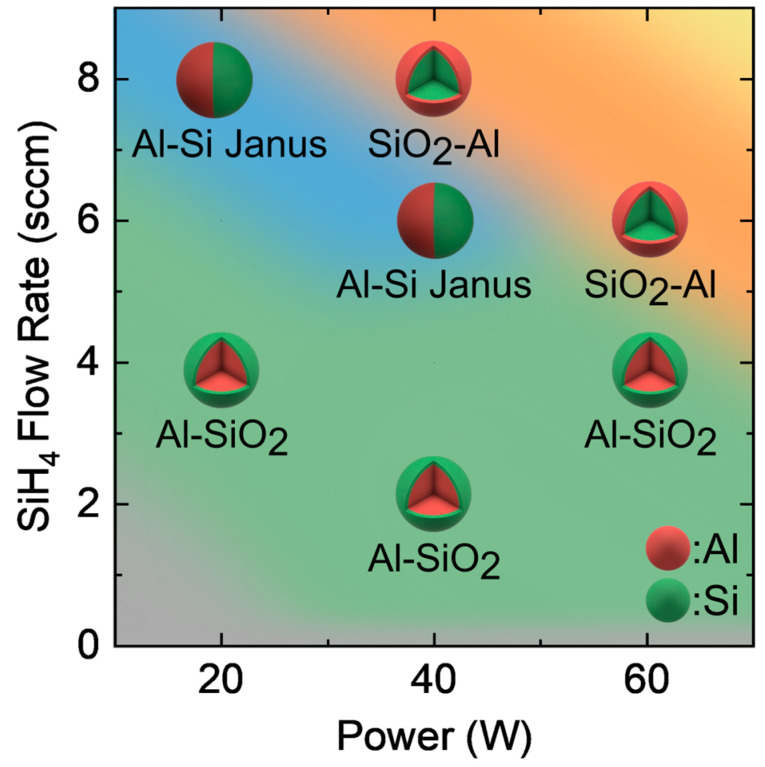
Qualitative approximation of particle characteristics with power and dilute SiH_4_ flow rates.

Analysis of the XRD patterns combined with insights from TEM and STEM-EDS elemental analysis was used to construct a qualitative diagram of the studied parameter space for which the following morphologies would expect to be dominant: (1) Al NP nucleation without a Si-containing shell when less than 2 sccm dilute SiH_4_, (2) Al–SiO_2_ core–shell NP formation in the range of 2–4 sccm dilute SiH_4_ at 20–40 W, (3) Janus Al–Si NPs at 8 sccm dilute SiH_4_ and 20 W or 6 sccm and 40 W, and (4) preferential c-Si nucleation without the formation of Al particles, such as Si–Al_2_O_3_ core–shells in combination with unmodified c-Si particles at dilute SiH_4_ flow rates at and beyond 4 sccm and 60 W. The qualitative map of observed particle heterostructures with the RF power and dilute SiH_4_ flow rate is presented in Figure 7.

The Al–SiO_2_ core–shell region is represented in green. The Si–Al_2_O_3_ region is denoted by orange in the top right. The Al–Si Janus particle region is shaded with blue (top left). The gray region (bottom left) indicates parameters that resulted in unmodified Al NPs. The regions where Si–Al_2_O_3_ formed are orange. The yellow region (top-right corner) indicates parameters that resulted in c-Si particles without detectable Al NPs in the bulk. Overall, this map summarizes how particle morphology can be changed by tuning either the dilute SiH_4_ flow rate or RF power.

### 3.3. Surface Analysis

XPS and FTIR were conducted to provide additional insight into the surface composition. XPS survey scans were taken for samples deposited at 40 W and 2–4 sccm dilute SiH_4_. These survey scans, found in Figure S4, were used to determine the surface composition. As shown in Table S1, the composition of the samples differs with the increase in the dilute SiH_4_ flow rate. As the flow rate increases from 2 to 4 sccm, the O at.% decreases from 59.7% to 51.3%, and the Si at.% increases from 1.1% to 5.2%. The trend of more Si incorporation in the sample with an increasing precursor flow rate was consistent with the findings from EDS.

FTIR spectra were collected to investigate the surface chemistry of NP samples, as shown in Figure S5. The spectra are baseline corrected and normalized to the O–H peak at ~3200 cm^−1^. Al–O bonding at ~690 cm^−1^ and Al–O–Al vibrational mode at ~540 cm^−1^ were present in all samples. Samples deposited at RF powers of 40 W and 60 W with 6 sccm dilute SiH_4_ showed Si-O-Si asymmetric stretching peaks and were observed at 1100 to 1150 cm^−1^ [50,51]. In all samples, peaks attributed to Si-O-Al were found at 1018 to 975 cm^−1^. These peaks shifted to slightly higher wavenumbers with increasing dilute SiH_4_ flow rates at 40 W, indicating a decreasing Al:Si ratio [51]. Overall, from FTIR and XPS survey scans, the sample surface species showed sensitivity to the RF power and dilute SiH_4_ flow rate.

**Figure 8:**
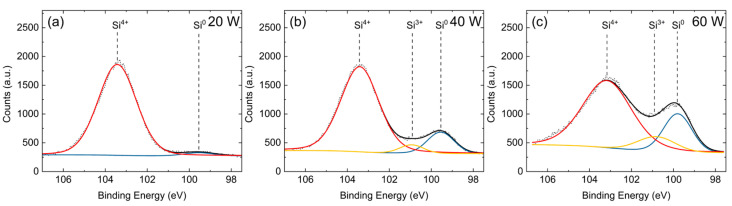
XPS high-resolution scans of the Si 2p peak with peak fittings for (**a**) 20 W, (**b**) 40 W, (**c**) 60 W, and 4 sccm dilute SiH_4_ samples. χ^2^ = 2.24, 2.95, and 2.92, respectively. The dotted line is the raw spectrum; red, yellow, and blue are the fitted peaks for Si^4+^, Si^3+^, and Si^0^, respectively; and black is the composite spectrum.

To better understand the Si surface layer, high-resolution scans of the Si 2p peak were measured for samples deposited at 20, 40, and 60 W and 4 sccm dilute SiH_4_, as shown in Figure 8. Each spectrum was deconvoluted into its contributions from Si-Si bonding, sub-oxide bonding (SiO_x_), and fully oxidized silica (SiO_2_). The binding energies and relative contributions of each are described in Table 2. The oxidation of the surface Si layer occurred under atmospheric conditions. The oxidation of a-Si in an air atmosphere at room temperature is understood to result in the Si 2p peak having several intermediate convoluted sub-oxide peaks [52]. For the 20 W sample, there are only two appreciable peaks, with most of the sample’s surface consisting of SiO_2_. As the plasma power increases, the sample surface becomes notably less oxidized, as seen in the decrease in the SiO_2_ peak, the emergence of the SiO_x_ peak, and the increase in the Si peak for both 40 W and 60 W samples. Altogether, this indicates a thicker, less oxidized Si surface layer with increased RF power. This is supported by the EDS, XRD, and FTIR results.

### 3.4. TGA and DSC Analysis

**Figure 9:**
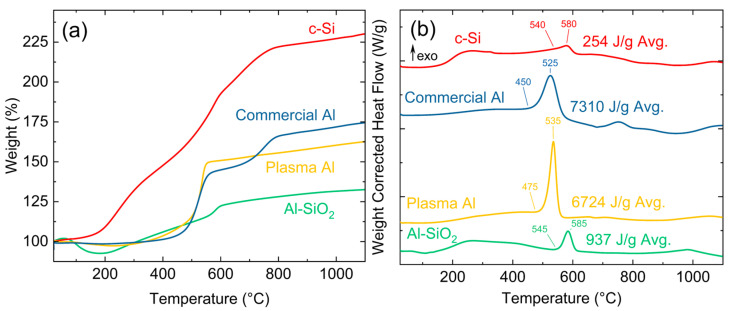
(**a**) TGA and (**b**) DSC results for NovaCentrix Al, unmodified Al NPs, Al–SiO_2_ core–shells deposited at 40 W and 3 sccm dilute SiH_4_, and c-Si samples.

TGA and DSC data were obtained and averaged over three or more trials in a synthetic air mixture for unmodified plasma-synthesized Al NPs, H-terminated c-Si, and Al–SiO_2_ core–shell samples deposited at 40 W and 3 sccm dilute SiH_4_. This condition was chosen since XRD pattern data indicated the crystalline phase in the bulk was FCC Al and the morphology of the NPs in the sample was an Al–SiO_2_ core–shell. The unmodified Al NPs were deposited at 60 W with no SiH_4_ added during synthesis. This condition resulted in unmodified crystalline Al NPs. The data were compared to measurements of commercially available NovaCentrix Al nanopowder (NovaCentrix, Austin, TX, USA) with a reported nominal size of 80 nm over the same testing conditions. In Figure 9a, the TGA results for the unmodified plasma Al NPs showed an average weight gain of 58.2% due to oxidation. In Figure 9b, DSC curves are shown, with the positive y-direction being exothermic. Integration of the exotherm peak was used to calculate the heat release per unit mass of material. For unmodified plasma Al NPs, there was an exotherm due to the oxidation of aluminum that corresponded to an average heat release of 6724 J/g, with an onset temperature of ~475 °C and a peak temperature of 535 °C. These oxidation onset and peak temperatures were also confirmed with the first derivative of the TGA curves plotted in Figure S6. NovaCentrix Al nanopowder, which had an average weight gain of 76.0%, showed similar rapid oxidative behavior to that of the plasma Al NPs. The exothermic reaction resulted in 7310 J/g heat release, with an exothermic onset temperature of ~450 °C and a peak temperature of 525 °C.

In the Al samples, three or more distinct stages were observed: (1) an initial weight loss of 1–3% with heating up to about 200–230 °C due to dehydration or loss of CO/CO_2_ on the surface, (2) rapid oxidation of Al at 525–535 °C, with a smaller secondary oxidation in the Novacentrix Al likely due to the melting and diffusion of the larger Al core, and (3) slow oxidation of remaining Al up to 1100 °C. This generally agrees with previously reported behavior for Al nanopowders under slow heating rates [53,54].

TGA measurements of Al–SiO_2_ NPs indicated an average weight gain of 33.0%. Although the overall weight % gain was similar to that observed for plasma-synthesized Al, the rate of oxidation was much slower. These samples showed a smaller exothermic reaction resulting in 937 J/g of heat release, which was likely due to the decreased mass fraction of Al with the addition of the SiO_2_ shell. The exotherm had an onset temperature of ~545 °C and a peak temperature of 585 °C. This oxidation temperature is 50 °C higher than that of the unmodified plasma Al NPs. This may be due to the additional passivation of the SiO_2_ shell. Computational modeling of Al–SiO_2_ during combustion showed that the number of Al–O and Si–O bonds in the simulated particle directly indicated the stability and reaction mechanism of the system during heating [42]. At low temperatures, Al–O and Si–O bonding remained constant, but at elevated temperatures, Al–O bonding increases, Si–O bonding decreases, and Si-Si bonds are formed, indicating oxidation of the Al resulting from the diffusion of O through the SiO_2_ shell. In this study, the Al–SiO_2_ samples appeared to have limited interaction between the core and shell materials. This is likely due to the high temperature of 1827 °C required for a reaction to occur with Al and SiO_2_ [55]. 

Similar to the Al–SiO_2_ samples, the c-Si samples exhibited steady weight gain with increasing time and temperature. c-Si had an average weight gain of 127%, which is consistent with previously reported values [56]. A small exothermic release of 254 J/g was measured with an onset temperature of ~540 °C and a peak temperature of 580 °C. Altogether, as shown in Table 3, the addition of Al to Si increased the exothermic behavior, but the Al–SiO_2_ material did not approach the recorded results for either commercial or plasma-synthesized Al NPs.

## 4. Conclusions

This work demonstrated control over a unique flow-through process utilizing an all-gas-phase capacitively coupled plasma to synthesize Al–SiO_2_ core–shell nanoparticles. Material characterization with STEM, EDS, XRD, XPS, and FTIR indicated a dependence on the applied power and dilute SiH4 flow rate to control the composition of the resultant nanoparticles. Al–SiO_2_ structures were confirmed with elemental maps, and EDS indicated a tunable range of the Al:Si ratio from 0.2 to 9.8. Although out of the scope of this study, future work in the Al–Si material system should consider using DFT, MD, or ReaxFF modeling to describe the evolution of the nanomaterial produced by the system under a wide range of plasma parameters.

Tuning limits were identified for Al–SiO_2_ core–shell heterostructures, as there was a tendency to form phase-separated material (Al–Si Janus particles, or Si–Al_2_O_3_ core–shell NPs) when either the RF power or dilute SiH_4_ flow rate was relatively high. FTIR and XPS analyses confirmed the strong influence of the applied RF power and dilute SiH_4_ flow rate on the nanoparticle surfaces as well, suggesting that the bonding environment and surface composition for all Al–Si heterostructures in this work can be modified by these two parameters to favor more or less surface oxide. TGA/DSC results showed that the peak exothermic release for the Al–SiO_2_ sample was 937 J/g, which was much lower than that of the unmodified plasma-synthesized Al NPs at 6724 J/g. Additionally, the Al–SiO_2_ sample demonstrated an oxidation temperature increase of 50 °C over plasma-synthesized Al NPs.

## Figures and Tables

**Table 1:** EDS (at.%) for regions of interest in Figure 3, Figure 4 and Figure 5.

Sample	C at.%	O at.%	Al at.%	Si at.%	Al:Si Ratio
Al–SiO_2_ (Figure 3a)	50.2	22.4	21.6	2.2	9.8
Al–SiO_2_ (Figure 3b)	35.1	37.7	10.8	11.2	1.0
Al–SiO_2_ (Figure 3c)	73.3	14.3	1.5	7.7	0.2
Al–Si Janus (Figure 4)	52.0	22.5	15.3	8.3	1.8
Si–Al_2_O_3_ (Figure 5)	53.4	20.7	3.4	20.9	0.2

**Table 2:** XPS high-resolution Si 2p peak fitting binding energies and relative contributions for 20 W, 40 W, and 60 W samples with χ^2^ = 2.24, 2.95, and 2.92, respectively.

	20 W		40 W		60 W	
	Binding Energy (eV)	Area (%)	Binding Energy (eV)	Area (%)	Binding Energy (eV)	Area (%)
Si	99.55	2.40	99.55	12.98	99.79	20.50
SiO_x_	-	0	100.94	4.47	100.82	10.06
SiO_2_	103.43	97.60	103.41	82.55	103.17	69.44

**Table 3:** TGA/DSC average results for NovaCentrix Al, unmodified Al NPs, Al–SiO_2_ core–shells, and c-Si samples.

Sample	Avg. Initial Mass (mg)	Weight % Gain (%)	Avg. Peak Exotherm (J/g)	Exotherm Onset Temp. (°C)	Exotherm Peak Temp. (°C)
c-Si NPs	2.585 mg	127%	254	540	580
Commercial Al NPs	3.994 mg	76%	7310	450	525
Plasma Al NPs	4.231 mg	58%	6724	475	535
Al–SiO_2_ NPs	8.186 mg	33%	937	545	585

## Data Availability

The raw data supporting the conclusions of this article will be made available by the authors upon request.

## Supplementary Materials

The following supporting information can be downloaded at https://www.mdpi.com/article/10.3390/nano15030237/s1, Figure S1, STEM-HAADF and EDS elemental maps for Al–SiO_2_ core–shell particles formed at (a) 20 W and 4 sccm dilute SiH_4_ (b), 50 W and 4 sccm dilute SiH_4_, and (c) 60 W and 3 sccm dilute SiH_4_; Figure S2, TEM and size distribution plots for core–shell samples (a) 20 W and 4 sccm SiH_4_, N = 205, (b) 50 W and 4 sccm SiH_4_, N = 204, and (c) 60 W and 3 sccm SiH_4_, N = 132; Figure S3, STEM-HAADF and EDS elemental maps for (a) Al–Si Janus particles deposited at 20 W with 8 sccm dilute SiH_4_; Figure S4, XPS surveys for the samples deposited at 40 W with 2, 3, and 4 sccm dilute SiH_4_; Table S1, XPS at.% for samples deposited at 40 W and 2, 3, and 4 sccm dilute SiH_4_; Figure S5, FTIR spectra for samples deposited at (a) 20, 40, and 60 W and 6 sccm dilute SiH_4_, and (b) 40 W and 0, 2, and 4 sccm dilute SiH_4_; Figure S6, the derivative of the TGA curves for plasma-synthesized crystalline Si, commercial Al NPs, plasma-synthesized Al NPs, and Al–SiO_2_ core–shell samples.

## Abbreviations

The following abbreviations are used in this manuscript:

Al NPs	Aluminum nanoparticles
AlCl_3_	Aluminum trichloride
Al–SiO_2_	Aluminum–silica core–shell nanoparticles
Ar	Argon gas
a-Si	Amorphous silicon
At.%	Atomic percent
CCP	Capacitively coupled plasma
c-Si	Crystalline silicon
DSC	Differential scanning calorimetry
EDS	Energy-dispersive X-ray spectroscopy
FTIR	Fourier-transform infrared spectroscopy
H_2_	Hydrogen gas
HAADF	High-angle annular dark field
RF	Radiofrequency
Si NPs	Silicon nanoparticles
Si–Al_2_O_3_	Silicon–alumina core–shell nanoparticles
SiH_4_	Silane gas
STEM	Scanning transmission electron microscopy
TEM	Transmission electron microscopy
TGA	Thermogravimetric analysis
Wt. %	Weight percent
XPS	X-ray photoelectron spectroscopy
XRD	X-ray diffraction
μ_g_	Geometric mean diameter
σ_g_	Geometric standard deviation
