# Supplementary material for: Aluminum–Silica Core–Shell Nanoparticles via Nonthermal Plasma Synthesis
Source: Nanomaterials (Basel). 2025 Feb 4;15(3):237. doi: 10.3390/nano15030237 (PMC11820144; doi:10.3390/nano15030237)
Supplement: Supplementary file 1 [file nanomaterials-15-00237-s001.zip › nanomaterials-3429721-supplementary.pdf]

Supporting Information

Aluminum-Silica Core-Shell Nanoparticles via Nonthermal Plasma Synthesis

Thomas Cameron[1], Bailey Klause[2], Kristine Loh[2], Uwe Kortshagen[1*]

[1] Department of Mechanical Engineering, University of Minnesota, Minneapolis, Minnesota 55455, United States
[2] Department of Chemical Engineering and Materials Science, University of Minnesota, Minneapolis, Minnesota 55455, United States
* Correspondence: kortshagen@umn.edu; U.R.K.

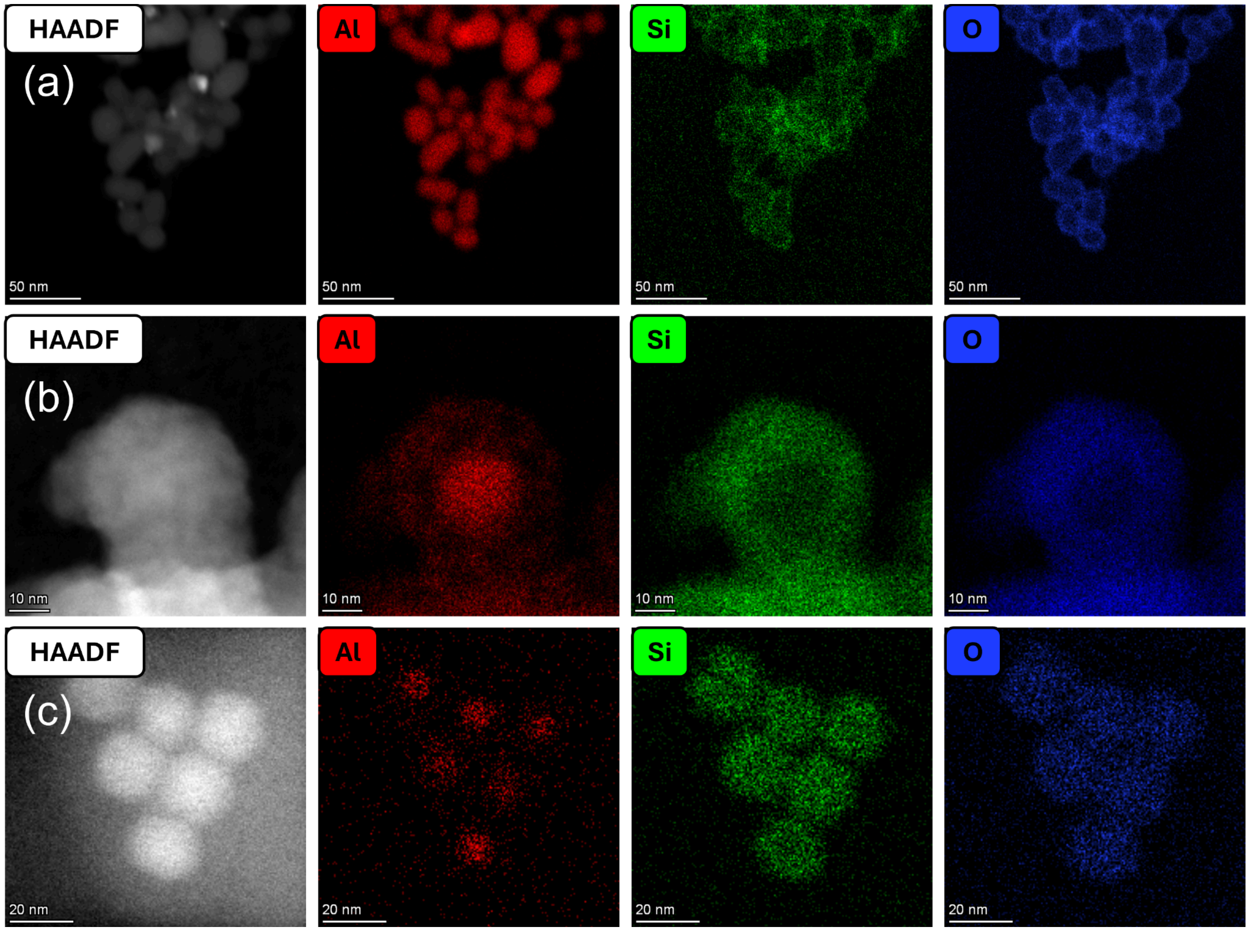

Figure S1. STEM-HAADF and EDS elemental maps for Al-SiO_2 core-shell particles formed at (a) 20 W and 4 sccm dilute SiH_4 (b) 50 W and 4 sccm dilute SiH_4, and (c) 60 W and 3 sccm dilute SiH_4.

Figure S1 shows the STEM-EDS elemental maps of samples made under core-shell forming conditions. STEM-EDS data within each sample indicated that a majority fraction of observed particles exhibited core-shell structure; however, in some samples, up to 10% remained unmodified Al NPs. The particles within the sample exhibited mostly spherical or

irregular ellipsoidal morphologies. In the conditions presented above, Si and O were localized to a shell structure and Al formed the core.

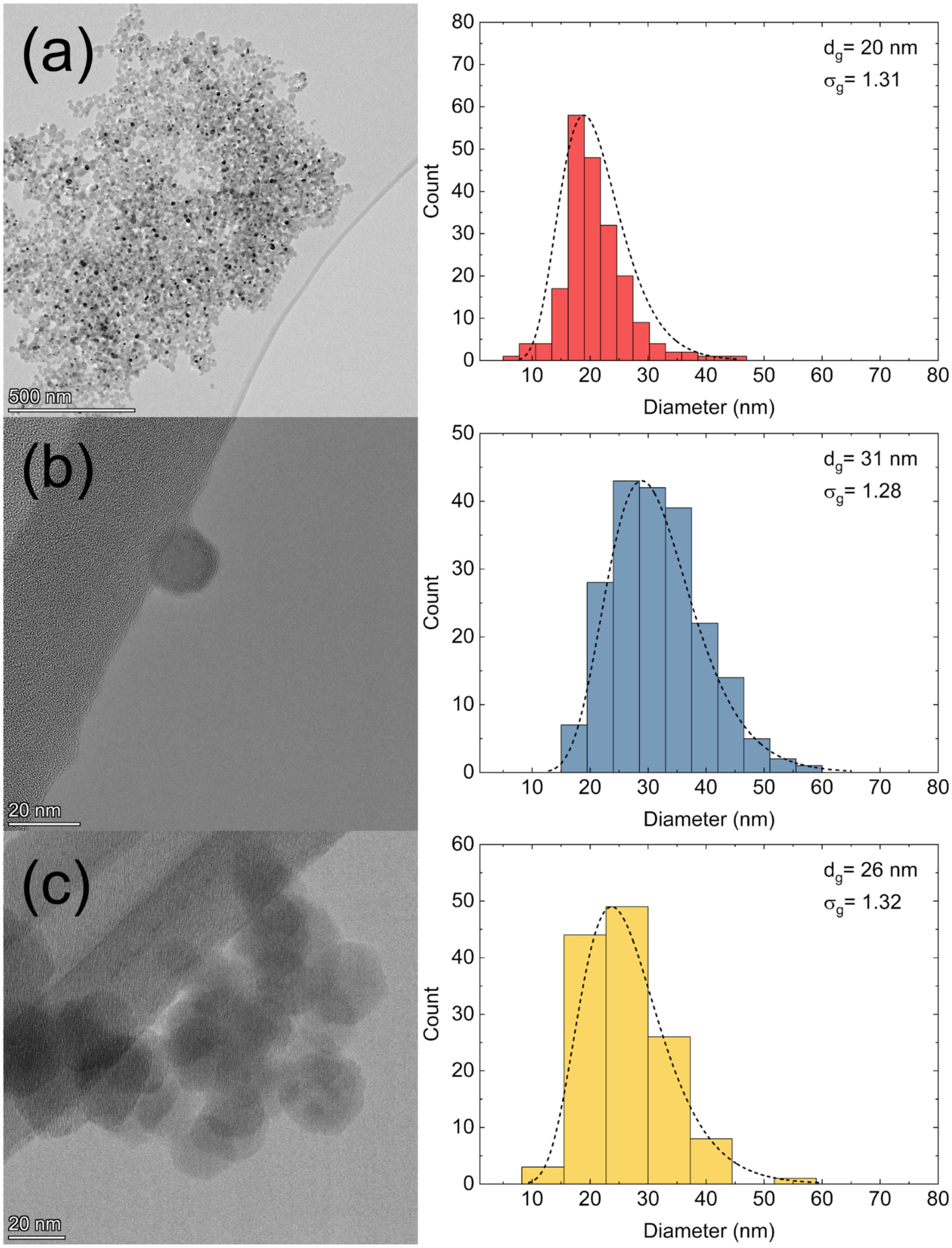

Figure S2. TEM and size distribution plots for core-shell samples synthesized at (a) 20 W & 4 sccm SiH_4, N=205, (b) 50 W & 4 sccm SiH_4, N=204, and (c) 60 W and 3 sccm SiH_4, N=132.

Figure S2 shows representative TEM images and particle size histograms fitted with lognormal distributions for three core-shell forming conditions. The geometric mean diameters and geometric standard deviations are given in the figure.

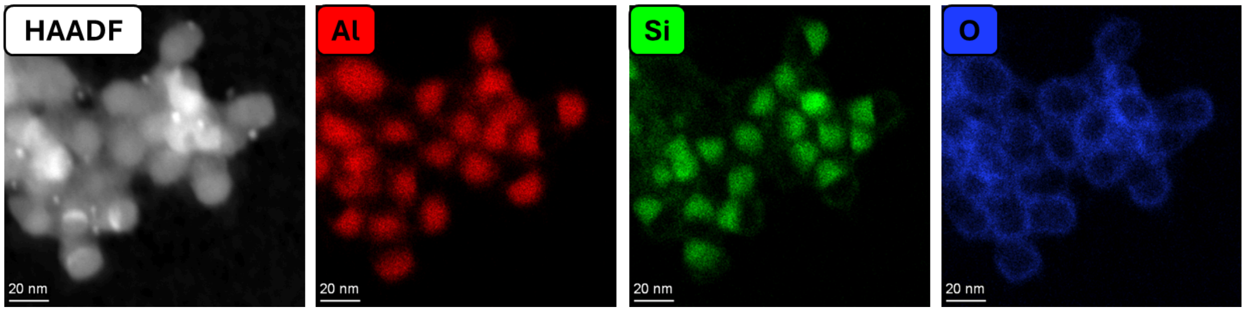

Figure S3. STEM-HAADF and EDS elemental maps for Al-Si Janus particles deposited at 20 W with 8 sccm dilute SiH_4.

Figure S3 shows the STEM EDS elemental maps of Al-Si Janus nanoparticles. These data indicated that an oxide shell exists on the particle surface, but not at the boundary between Al and Si. The particle morphology is irregular and ellipsoidal under these conditions.

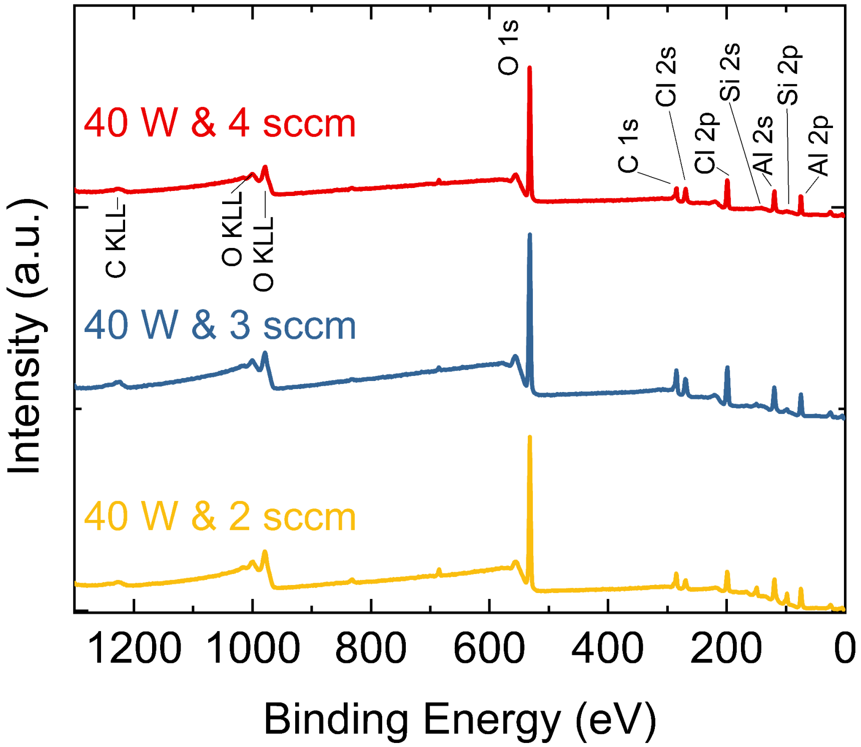

Figure S4. XPS Surveys for the samples deposited at 40W with 2, 3, and 4 sccm dilute SiH_4.

Figure S4 shows the XPS survey scans for samples deposited at dilute SiH_4 flow rates of 2, 3, and 4 sccm. Table S1 shows the elements' at.% from each survey.

Table S1. XPS at.% for samples deposited at 40 W and 2, 3, and 4 sccm dilute SiH_4

Sample	O at.%	Al at.%	Si at.%	Cl at.%
40 W & 2 sccm dilute SiH_4	59.7	14.6	1.1	4.9
40 W & 3 sccm dilute SiH_4	50.8	13.9	2.3	8.9
40 W & 4 sccm dilute SiH_4	51.3	15.8	5.2	8.6

Adventitious carbon was present in all samples and accounted for the remaining at.% at the surface. A primary surface contaminant was Cl which was attributed to using $AlCl_3$ as a precursor material for Al NP formation. The high at.% of O results from sample exposure to the atmosphere before characterization. Another contributor to the significant fraction of O in the survey is that a minority of the particles within the sample are unmodified Al NPs that oxidized readily when exposed to air before characterization.

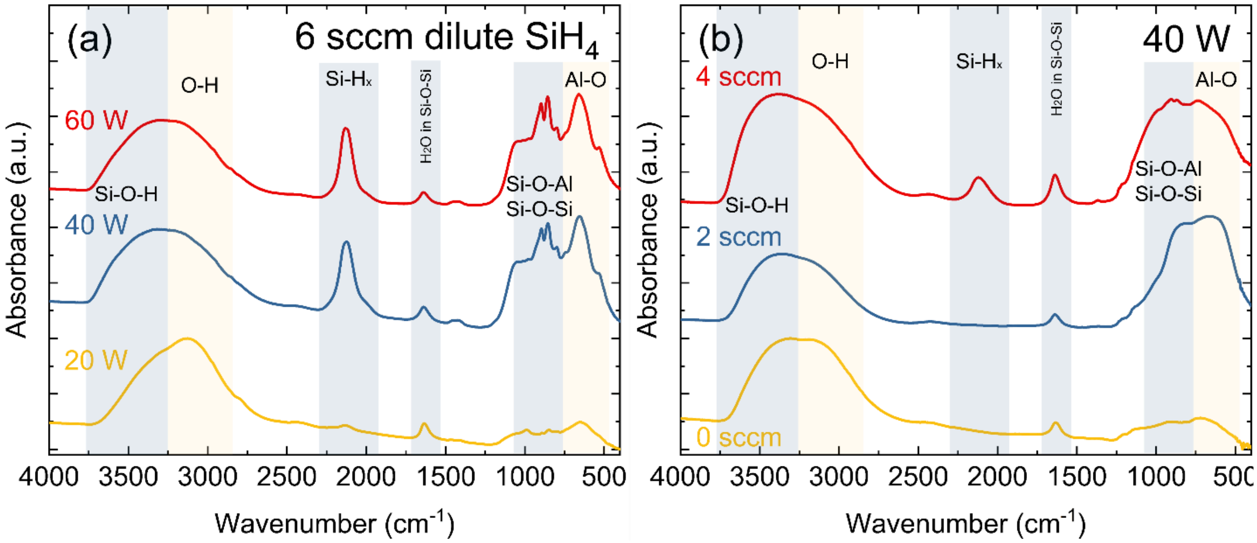

Figure S5. FTIR spectra for samples deposited at (a) 20, 40, 60 W and 6 sccm dilute SiH_4, and (b) 40 W and 0, 2, 4 sccm dilute SiH_4.

FTIR spectra are shown in Figure S5. Highlighted bands are indicated to summarize the peak assignments relevant to Al and Si in orange and blue, respectively. Figure S5(a) shows FTIR spectra for a constant dilute SiH_4 flow rate of 6 sccm and RF powers of 20, 40, and 60 W. An increasingly dominant Si-H_x peak at ~2100 cm^{-1} was identified which is associated with silicon-hydrogen surface groups. The Si-H_x peak also emerged at 40 W and 4 sccm dilute SiH_4 in

Figure S5b. Peak shifting in the O-H region from ~3200 cm^{-1} to ~3300-3500 cm^{-1} was observed with increasing power or dilute SiH_4 flow rate. These results indicate that the presence of Si and Si-H_x in c-Si increased with RF power and dilute SiH_4 flow rate.

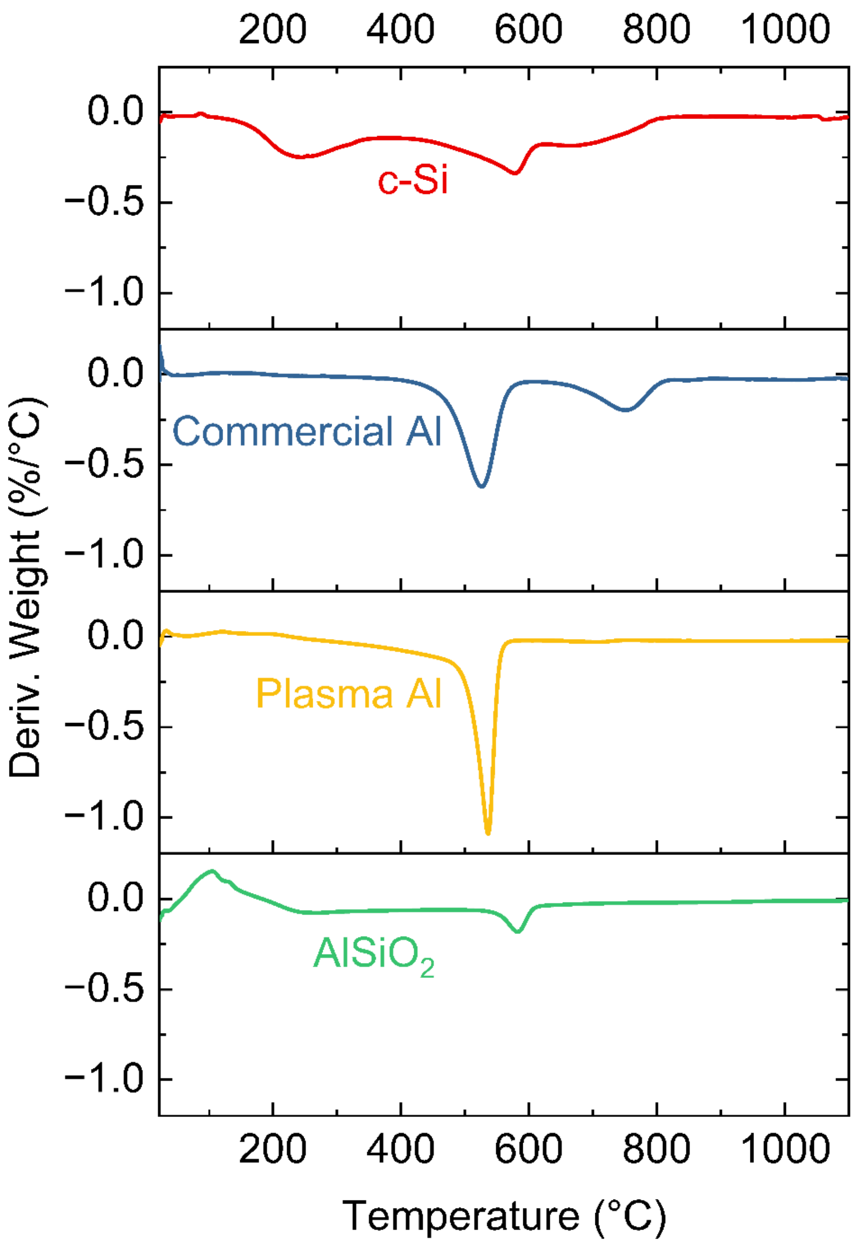

Figure S6. The derivative of TGA curves for plasma-synthesized crystalline Si, commercial Al NPs, plasma-synthesized Al NPs, and Al-SiO_2 core-shell samples.

Figure S6 shows the derivative of weight with temperature versus furnace temperature. Rapid weight gain is observed in the commercial and plasma Al NPs at 530℃. This aligns with heat release from DSC data for these samples. The c-Si and Al-SiO_2 samples had higher onset temperatures for rapid weight gain at approximately 570℃.
